# COVID-19 vaccination in patients with cancer: Opportunities and challenges

**DOI:** 10.3389/fonc.2022.1029325

**Published:** 2022-11-08

**Authors:** Zahraa Haleem Al-qaim, Hasanain Kamil Hasan Owadh, Sarah A. Ali, Alaa S. Hussein, Thamer Ramadhan Ameen, Ayshan Kolemen, Ghassan A. Washi, Abduladheem Turki Jalil

**Affiliations:** ^1^ Anesthesia Techniques Department, Al-Mustaqbal University College, Babylon, Hilla, Iraq; ^2^ Department of Pharmacy, Al-Mustaqbal University College, Babylon, Hilla, Iraq; ^3^ Department of Medical Laboratory Techniques, Al-Mustaqbal University College, Babylon, Hilla, Iraq; ^4^ Radiological Techniques Department, Al-Mustaqbal University College, Babylon, Hilla, Iraq; ^5^ Department of Law, Al-Mustaqbal University College, Babylon, Hilla, Iraq; ^6^ Department of Dentistry, Al-Mustaqbal University College, Babylon, Hilla, Iraq; ^7^ Department of Nursing, Al-Mustaqbal University College, Babylon, Hilla, Iraq; ^8^ Medical Laboratories Techniques Department, Al-Mustaqbal University College, Babylon, Hilla, Iraq

**Keywords:** SARS-CO-V-2, COVID-19 vaccination, cancer, solid tumor, hematologic cancer

## Abstract

The rapid spread of the SARS-Cov-2 virus, the increase in the number of patients with severe COVID-19, and the high mortality rate created the basis for the production of safe and effective vaccines. Studies have confirmed the increased risk of severe Covid-19 disease and mortality in cancer patients. It is logical that cancer patients should be the first to receive the primary vaccination and the booster vaccine for Covid-19. Since studies related to cancer patients and the effectiveness of existing Covid-19 vaccines have not been widely conducted, there are significant uncertainties about the effectiveness of the vaccine and the level of humoral and cellular immune responses in these patients. As a result, the possible risks and side effects of existing vaccines are not clear for patients with different cancers who are undergoing special treatments. In this study, we will discuss the effectiveness and safety of existing vaccines on cancer patients. In addition, we highlight factors that could affect the effectiveness of vaccines in these patients and finally discuss opportunities and challenges related to vaccination in cancer patients.

## Introduction

Coronavirus disease 2019 (COVID-19), caused by the severe acute respiratory syndrome coronavirus 2 (SARS-CoV-2) (1), has caused more than 5 million deaths worldwide (2). About 80% of patients with covid-19 either do not show symptoms or the symptoms are weak. But patients with severe symptoms show some symptoms, including excessive inflammation, and immune system dysfunction, that is very similar to a malignancy (3). Cancer patients are more exposed to worse consequences of viral infections (4). COVID-19-related mortality in patients with underlying diseases such as cancer patients is considerable though the symptoms may not be as they are expected (5, 6). Moreover, a study showed that almost 20% of these patients may suffer from an asymptomatic disease was evident only in serological assessment (7). Older people, and patients taking immunosuppressive drugs (cancer patients) have more severe symptoms when they get COVID-19 (8). In addition, the death rate in cancer patients with covid-19 is higher compared to patients without cancer (9). Accordingly, it seems very necessary to protect cancer patients from contracting COVID-19. Although using a mask and maintaining social distance reduces the risk of being exposed to the SARS-CoV-2 virus, it can still impose negative psychological effects on a person. But using the vaccine can have more tangible positive effects.

Among all cancer patients, patients who have received stem cells, patients with blood-related malignancies, and patients with lung cancer are at higher risk (10–12). Over 19 million new cancers have been recorded in the GLOBOCAN database by 2020 worldwide. This statistic is a fundamental challenge for people who want to care for cancer patients against the SARS-CoV-2 virus. Health care providers are forced to implement preventive strategies such as quarantine or vaccination on this number of cancer patients. Although vaccination is a viable strategy to prevent contracting COVID-19 in cancer patients, there are still misconceptions about vaccination in cancer patients, especially during treatment with immunosuppressive drugs. The aim of this study was to identify appropriate recommendations for vaccination for cancer patients and to achieve an appropriate immune response during immunosuppressive therapy. Also, we examine the time limits of using vaccination, and the appropriate response to the challenges in cancer management in the Corona era.

## SARS-Cov-2 and patients with cancer

Cancer patients must go to medical centers to follow the treatment process, and they are more exposed to SARS-CoV-2 infection, so they are susceptible to contracting COVID-19. Also other treatments, such as gene therapy, radiotherapy, and chemotherapy used to treat cancer patients (13), often suppress patients’ immune systems. These factors make the symptoms of COVID-19 severe in cancer patients. Therefore, disease management will be very challenging.

### Hematologic cancer

Patients with hematologic malignancies are susceptible to COVID-19 due to dysfunction of the humoral and cellular immune systems, cytotoxic chemotherapy, and old age (14, 15). Studies showed that immune response (seroconversion) rates following COVID-19 vaccination in patients with underlying diseases such as cancer patients are compromised in older patients, those with hematological malignancies, and chemotherapy receivers (16, 17). The results of a study showed that patients with blood malignancies had the highest mortality rate (37%) among all types of cancer patients with Covid-19. Interestingly, 55% of lung cancer patients died of Covid-19 disease (14). Repetition of these results has been observed in other studies (18–21). In children with cancer and Covid-19, the mortality rate was less than 5%, while in people over 60, the mortality rate was close to 40%. Older age appears to increase the risk of COVID-19 in these cancer patients (22).

The type of cancer, the time of diagnosis, the stage of cancer, and the type of treatment are factors that play an important role in increasing the risk of severe COVID-19 in patients with blood malignancies. The most common malignancies were acute myeloid leukemia (AML), (33%), non-Hodgkin’s lymphoma (27%), and myeloma or amyloidosis (16%) (23). There was a difference in mortality rates between different cancers, so patients with acquired bone marrow failure and AML infected with the SARS-CoV-2 had the highest mortality rate (24). Patients with AML and COVID-19 have a worse prognosis than other blood malignancies suffering from COVID-19 (18, 25). However, the cause of this issue has not been determined. Patients with hematologic malignancies have immunodeficiency, in patients with blood malignancies (including AML and multiple myeloma) T cell dysfunction is very evident (26, 27). Despite the immunodeficiency in these patients, the rate of response to infection can be very significant. Evaluation of this defect and response rate to SARS-CoV-2 infection is necessary to better understand the pathogenic mechanisms in patients with hematologic malignancies.

On the other hand, the fear of contracting Covid-19 can reduce the number of cancer patients who go to the relevant centers for treatment and cause irreparable damage to the patients (28). Administration of cytotoxic chemotherapy in cancer patients shows different degrees of immune system suppression based on duration, the intensity of suppression, and type of suppression. These factors make the relationship between cytotoxic chemotherapy and its effects different in patients with cancer and Covid-19. The results of various studies support the above statements as some studies report no association (22, 29, 30) while others support the association between anti-cancer therapies and the severity of COVID-19 (18, 31, 32). Nevertheless, some studies have defended the protective effect of cancer-based therapies against COVID-19 (33, 34). SARS-CoV-2 aggravates the symptoms of COVID-19 by increasing the production of cytokines. Thus, JAK and BTK inhibitors can improve COVID-19 symptoms by targeting cytokine secretion (35, 36). A group examined risk factors for survival in patients with myeloproliferative neoplasms (MPN) and COVID-19. The results showed that people treated with ruxolitinib had a high mortality rate. Interestingly, they found no association between ruxolitinib treatment and overall mortality but found that there was an increase in mortality after stopping the drug (33). Hong Jin et al. examined a 39-year-old patient with a medical history of non-Hodgkin’s lymphoma and chronic lymphocytic leukemia with symptoms of Covid-19. Although the patient had previously received chemotherapy with R-CHOP and oral chlorambucil (10 mg/m2) for cancer treatment, oxygen supplementation reduced respiratory symptoms (37). All these findings show that there are currently no precise mechanisms of the effects of SARS-CoV-2 in blood patients. Extensive studies with large statistical populations on blood cancers can shed light on many unknowns.

### Solid tumors

In patients with Covid-19 without cancer, the symptoms subside within 7-14 days. But for patients with solid tumors (one month), hematologic malignancies (about 2 months), and patients with hematologic malignancies who have had stem cell transplants, it takes more than 60 days for recovery (38, 39). This increase in the recovery period is due to the fact that cancer patients have a weak immune system due to dysfunction of immune cells and cannot function properly against infection, which is more common in hematologic malignancies (30, 40). But in general, the main reason for the suppression of the immune system by direct methods (bone marrow transplantation) (41) and indirect (cytotoxic chemotherapy) therapy (42). The results of a retrospective cohort study showed Lung cancer was the most common type of cancer. 53.6% of patients showed severe symptoms of COVID-19 and eight died. If cancer patients are treated with anticancer drugs 14 days before SARS-CoV-2, the consequences of COVID-19 are significantly more severe [hazard ratio (HR) = 4.079, 95% confidence interval (CI) 1.086-15.322, P = 0.037] (43). In another similar study by Yang et al. Similar results were obtained for 52 cancer patients whose COVID-19 disease was confirmed. Lung cancer was the most common cancer, and about 21% of cancer patients died (44). Tian et al. monitored two asymptomatic Covid-19 patients who underwent lobectomy for lung cancer. The first patient was a 73-year-old man with high blood pressure who developed Covid-19 after surgery but recovered after appropriate treatment. The second patient was an 84-year-old woman with hypertension and diabetes who developed Covid-19 after lobectomy. However, supportive treatments were not helpful and the patient died less than a month after the lobectomy (45).

The immune status of solid tumor cancer patients after primary vaccination is dynamic and not all of them show an adequate immune response. Therefore a third booster dose seems necessary for insufficient responders (46). A third dose can boost anti-Covid-19 immunity in cancer patients, but the duration of protection is difficult to predict and requires further study (47). In patients with MM after vaccination with the BNT162b2 mRNA vaccine, focusing on their response before and at 1 month after the fourth vaccination. Booster vaccination with the BNT162b2 results in a substantially improved humoral response against SARS-CoV-2 in patients with MM (48).

The importance of the risk of severe COVID-19 in cancer patients should not be underestimated. However, some cancer patients have mild symptoms of COVID-19, so worrying and stressing in these patients does not seem like a good idea. A married couple with symptoms of Covid-19 was admitted to the emergency room at the same time. The woman recently underwent fulvestrant and abemaciclib chemotherapy for recurrent breast cancer. The husband was healthy but had a history of high blood pressure. Both Covid-19 patients received similar treatment. The woman left the hospital on the sixth day despite suffering from a benign immunodeficiency. In contrast, her husband recovered slowly and needed intensive care (49). Based on this, it can be concluded that SARS-CoV-2 is not only affected by a weak immune response, but also by other factors. Therefore, identifying immune mechanisms after SARS exposure to CoV-2 is essential. These can improve emerging therapies.

## Types of vaccines used to prevent Sars-cov-2

**Figure 1:**
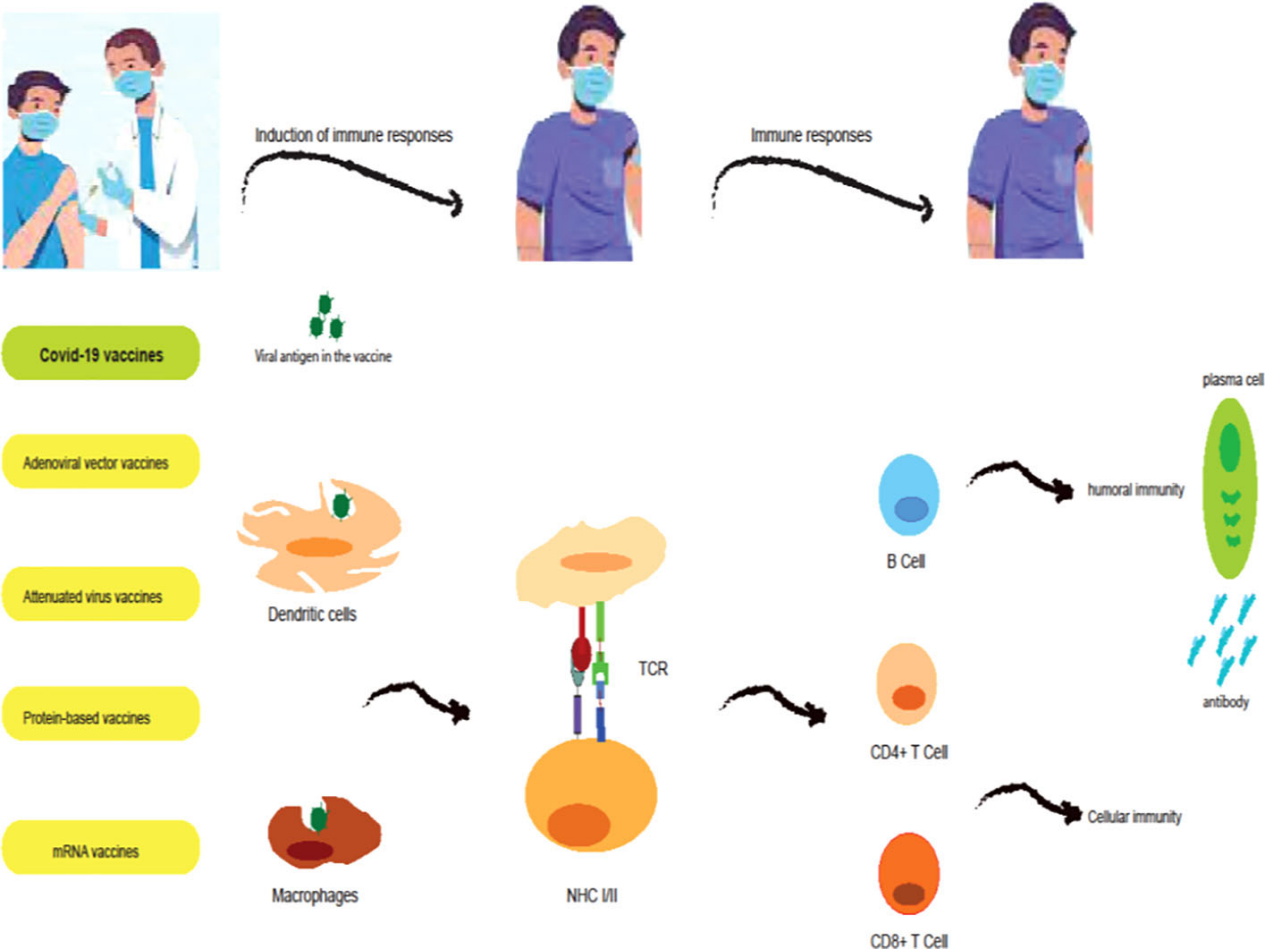
Induction of immune responses by COVID-19 vaccines. mRNA vaccines (BNT162b2 and mRNA-1273 vaccines) are a modified version of the spike protein which is translated by ribosomes, secreted by the bystander cell, and in turn taken up and processed by antigen-presenting cells (APCs; dendritic cells (DCs)). Adenoviral vector vaccines (ChAdOx1, Ad26.COV2.S, and Sputnik V vaccines) contain cDNA encoding a full-length spike protein. cDNA is transported to the nucleus where it is transcribed to mRNA and subsequently translated into spike protein in the cytoplasm. This spike protein is then taken up and processed by APCs. Protein-based vaccines (NVX-CoV2373 vaccines) consist of the spike protein and an adjuvant which is directly processed by APCs. Attenuated virus vaccines (CoronaVac and BBIBP-CorV vaccines) contain whole inactivated virus particles and adjuvants which APCs directly process. In the lymph nodes, APCs will present processed peptides and thus activate T cell responses and B cell responses, and antibody responses.

Humoral and cellular immunity protects the host body against viral infections (50). In humoral immunity, B lymphocytes block the entry of the virus into host cells by producing antibodies. Cytotoxic macrophages and T lymphocytes are involved in cellular immunity by killing infected cells. Therefore, vaccines should aim to induce specific antigens to form a protective memory to fight infection, so that the immune system can respond better to the infection after receiving the vaccine. Vaccination is one of the most important ways to prevent viral diseases such as SARS-CoV-2. The importance of vaccination in reducing mortality and reducing the economic burden on society and governments in the COVID-19 epidemic was very significant. The pandemic of COVID-19 in the whole world caused the efforts to develop a suitable vaccine to increase, which resulted in the production of more than ten suitable vaccines. To use these vaccines, their effectiveness in terms of safety, efficacy, and quality was evaluated and confirmed. However, the side effects of some vaccines need to be identified more carefully. It must be determined which vaccines are suitable for cancer patients and those with weakened immune systems (**Figure 1**).

### Live attenuated vaccines

Live vaccines are made using a weak form of a virus or a virus that has lost its ability to reproduce. The safety created by live vaccines is usually long-lasting, and only one dose can cause strong immunity. However, they are associated with a higher risk of developing symptomatic disease, especially in immunocompromised individuals (51, 52).

### Inactivated vaccines

Use physical or chemical methods to inactivate viruses in inactivated vaccines. Although inactivated vaccines do not cause infection, they do not induce severe cellular immunity, resulting in shorter immunity in the person using it (51). In addition, the production of these vaccines has time constraints because they must be cultured and inactivated on a large scale. Due to the fact that in such vaccines auxiliary substances are used. Therefore, they can cause limited side effects, especially at the injection site and exacerbate the inflammation. However, no severe associated side effects have been reported (53).

The SARS-CoV-2 passive vaccine, BBIBP-CorV, was administered in two doses two weeks apart and was well tolerated. Production of neutralizing antibodies to prevent covid-19 disease was induced after six weeks in vaccine recipients. In phase I/II trials, it did not cause any serious side effects, but had lower levels of neutralizing antibodies than recovering patients and low T cell responses (54). Zhang et al. tested a passive CoronaVac vaccine against COVID-19 for safety, tolerability, and immunogenicity. A decrease in primary immunity was observed within 28 days after injection in all participants (who received at least one dose of the drug). Overall, CoronaVac was well tolerated and was able to provide humoral immunity against SARS-CoV-2. However, there was no evidence that the vaccine elicited a cellular immune response. They only reported immune response data for healthy adults (55). Therefore, it should be used with greater caution in patients with comorbidities such as cancer and the elderly.

### Viral vector vaccines

Viral carrier vaccines consist of a transmissible virus (eg, adenovirus) with selected antigens from the target pathogen that are obtained by recombinant engineering. After inoculation, the engineered virus infects the host cells and leads to the expression of the virus antigen. These vaccines are very effective in promoting both humoral and cellular immunity, but their effectiveness can vary from person to person. Because most humans already have neutralizing antibodies against viral vectors, these antibodies can bind to the vectors and reduce the effectiveness of the vaccine, depending on the degree of binding. In addition, viral vectors can induce tumorigenesis in the user (56).

ChAdOx1 nCoV-19 (AZD1222) is a recombinant chimpanzee adenovirus vector vaccine containing the SARS-CoV-2 protein. Random analysis of randomized and controlled trials was performed for this vaccine. The results showed in participants who received two standard doses, vaccine efficacy was 62.1% in the ChAdOx1 nCoV-19 group, and in participants who received a low dose followed by a standard dose, efficacy was 90.0%. In the vaccinated group, spike-specific T cell responses peaked on day 14. Anti-spike IgG responses increased until day 28, and increased after the second dose. Age and dose received were two factors that changed the effectiveness of the vaccine from 62% to 90%. Side effects were somewhat balanced among the study groups. Only one case of transverse myelitis was observed following vaccination with AZD1222 (57, 58).

Ad26.COV2.S is a recombinant vector vaccine that encodes the SARS-CoV-2 spike (s) protein. Single-dose inoculation is sufficient to treat severe disease (59). The transient results of phase I/IIa showed that the most common systemic side effect was fever. The appropriate immune response was more in young people than in old people. More than 80% of patients had T helper 1 cell response, however, less than 65% of T cell responses were cytotoxic. The advantages that encourage the use of this vaccine are, firstly, that the use of a single dose is sufficient to produce immunity, and secondly, that it can be stored in a refrigerator (60). Nevertheless, the association between the Ad26.COV2.S vaccine and the onset of Guillain-Barré syndrome is highlighted in one study (61). It must be determined whether the main cause of infection is SARS-CoV-2 or vaccination. Which stimulate autoantibodies against peripheral nerve components. Can further stimulation of cellular immunity lead to severe side effects? These are questions that need to be answered convincingly.

### Nucleic acid vaccines

The best way to deal with emerging diseases such as SARS-CoV-2, which affects the world, is to expand vaccination. However, the vaccine must be produced in large quantities and more rapidly and be effective against the epidemic. Nucleic acid-based vaccines fall into two categories: DNA and RNA vaccines are a more promising choice because mRNA vaccines have the potential for rapid development with high efficacy and low cost (62). The first transfer of RNA and DNA for protein production was done about 32 years ago. A group of scientists was able to transfer mRNA reporter genes to mouse cells, resulting in protein production observed in mouse muscle for at least 2 months. This experiment became the basis for the development of mRNA vaccines (63).

BNT162b2 and mRNA-1273 are two important COVID-19 mRNA vaccines that have been approved. The BNT162b2 vaccine is an mRNA-based vaccine that uses the entire SARS-CoV-2 spike protein. It is produced by Pfizer-BioNTech against COVID-19 (64). The results of the studies showed that the titers of neutralizing antibodies and cellular immune responses are strong (65, 66). The third phase clinical trial was published by Pollack et al. The results of their study showed that 21,720 people had received the BNT162b2 vaccine. 95% of people 16 years of age or older are protected against SARS-CoV-2. However, mild side effects such as headache, fatigue, and mild to moderate pain were evident at the injection site (66).

mRNA-1273 is an mRNA vaccine against Covid-19 developed by Moderna. This vaccine is protected by nanoparticles and produces neutralizing antibodies by producing a part of the SARS-CoV-2 protein. The vaccine was studied in animal models of non-human mammals. The results showed that the vaccine enhances the responses of Th1 helper T cells (type 1). However, T cell responses were undetectable (67). The first phase clinical trial (ClinicalTrials.gov, NCT04470427) was a randomized, placebo-controlled, observational trial that showed 94.1% efficacy in vaccinated individuals 18 years of age and older (68).

### Protein subunit vaccines

In these vaccines, certain parts of the studied pathogen are used, such as proteins, sugars, and capsids in order to create a strong immune response. For SARS-CoV-2, the spike (s) protein, which is the major outer surface protein, may receive more attention because it plays a pivotal role in infiltrating host cells and can induce T-cells and antibodies (69). Because it is produced under laboratory conditions, the degree of infection with the virus is impossible. However, these vaccines are usually given in combination with adjuvants to produce appropriate and strong responses, resulting in increased costs and mild side effects (70, 71).

NVX-CoV2373 was evaluated for preclinical evaluation in mice and baboons. The results showed that in mice, low-dose NVX-CoV2373, combined with a saponin-based matrix-M adjuvant, produced antibodies that bound to the hACE2 receptor and blocked these receptors, protecting against SARS-CoV-2. It also produces T cells (CD4+ and CD8+), and B cells in the spleen. In baboons, low-dose levels of NVX-CoV2373 with Matrix-M were also highly immunogenic, and anti-S antibodies blocked the receptors. As a result, SARS-CoV-2 cannot penetrate host cells (72). The vaccination schedule for NVX-CoV2373 consists of two doses of intramuscular injection over 21 days. Phase I/II clinical trials showed that the use of NVX-CoV2373 in adults 18 to 59 years of age induces anti-S IgG protein and neutralizing antibodies. It also protects the user against infection by inhibiting the binding of SARS-CoV-2 to the hACE2 receptor (73).

## COVID-19 vaccine and patients with weakened immune systems

Immune system disorders and the use of immunosuppressive drugs are factors that weaken the immune system. These people are more susceptible to infectious diseases, especially COVID-19. They will have more severe consequences than COVID-19, including hospitalization and death. Even a reduction in the appropriate immune response to COVID-19 vaccination is to be expected in immunocompromised individuals compared with immunocompromised individuals.

In some diseases with COVID-19, such as high blood pressure and diabetes, severe disease outcomes and mortality increase (74). In type 2 diabetes, an increase in the regulation of synthesis is observed in proinflammatory molecules with low-grade inflammatory activity. This process stimulates immune cells for a long time and leads to immune imbalance (75). In addition, ACE2 is overexpressed in pancreatic islet cells, so SARS-CoV-2 is more likely to penetrate these cells, leading to severe blood glucose instability in diabetics and inflammatory imbalances (76). As a result, diabetic patients show more severe consequences after developing SARS-CoV-2. In clinical trials of SARS-CoV-2-related vaccines, healthy individuals without comorbidities, including diabetes, were included in the study. As a result, there is no conclusive evidence to suggest vaccine side effects and depth of protection in people with diabetes. In this context, clinical decision-making is largely evidence-based, as large-scale studies are limited. In one study, antibody levels in diabetics with healthy were evaluated three weeks after inoculation with the BNT162b2 vaccine. The results showed that diabetics had 4.42% fewer neutralizing antibodies than non-diabetics (77). Similar results were observed in a study conducted in Turkey (78). Although the production of neutralizing antibodies is lower in diabetics, this should not cast doubt on the vaccination of diabetics.

Multiple sclerosis (MS) is a chronic disease of the central nervous system (CNS) that causes the destruction of nerve cells by autoimmune reactions in CNS tissues (79). Various environmental factors can stimulate immune cells to attack nerve cells and cause MS (80). Yang et al. (81) examined the brain tissue of eight Covid-19 patients. The results of their study showed that SARS-CoV-2 peripheral infection inflames brain barrier cells such as the choroid plexus. In the brains of Covid-19 patients, choroidal cells secrete chemokines into the brain parenchyma that activate inflammatory mechanisms, thereby damaging brain tissue, suggesting that COVID-19 may be partially Repeat the pathological processes of various CNS diseases. Such cases have been observed in the mechanism of MS.

DMTs used to treat MS patients affect immune responses. However, the effect of DMT on COVID-19 in patients with MS is not well understood. The results of the studies are contradictory. One study found that the use of DMT in MS patients did not increase the risk of COVID-19 (82). In another study, the use of DMT, such as ocherlizumab or rituximab, was found to reduce the number of B cells in MS patients, making them more susceptible to severe Covid-19 disease (83). These data are preliminary and there is no pathophysiological relationship between B cell reducing agents and acute respiratory distress syndrome (ARDS) (84). Given that DMT can affect immune cells, the performance of vaccines in these patients will be confusing. SARS-CoV-2 infection can certainly cause recurrence or exacerbation of MS symptoms, vaccination is essential to protect against MS (51). One study found that some DMT drugs, including glatiramer acetate and triflunomide, could reduce the effectiveness of the rabies vaccine. However, vaccines can elicit a limited immune response (85). Interestingly, a study found that HPV vaccination could even reduce the risk of MS (86). Because live virus vaccines are contraindicated in patients with MS, DNA-RNA vaccines can be used in patients with MS whose immune systems are weakened (87). However, more studies are needed to help guide treatment strategies and optimize success with vaccination protocols. Further studies should also shed light on the positive and negative effects of vaccination on the recurrence and progression of neurological diseases, including MS.

## The role of cancer treatment in vaccination

Immunosuppression and the use of systemic therapy to treat cancer can have severe consequences for COVID-19 in cancer patients. Although extensive vaccination programs have been considered to reduce the severe symptoms of SARS-CoV-2 infection, concerns about the use of current vaccines in cancer patients are evident. These concerns are more pronounced in patients with blood malignancies than in those with solid cancers due to specific conditions. For patients diagnosed with hematologic malignancy in the last 5 years, an increased risk of death of at least 2.5-fold and for other cancers at least 1.2-fold has been observed (88). Therefore, prompt action for vaccination is necessary and important according to the vaccination recommendations in cancer patients.

### Cytotoxic chemotherapies

Cytotoxic chemotherapies seal the cell cycle by interfering with DNA synthesis. Lymphocytes proliferate more rapidly in the presence of antigens, so these treatments suppress their proliferation (89). However, inhibition of lymphocyte proliferation is not complete, so vaccines can elicit an immune response during cytotoxic chemotherapy. In patients with acute lymphoblastic leukemia, whose immune system is not functioning properly due to the nature of the disease and the use of the desired treatments, it can produce an immune response after vaccination. However, the amount of immune responses produced for different vaccines varies (89, 90). A study of patients with solid tumors and malignant lymphomas who received mild to moderate chemotherapy found that after a single dose of influenza vaccine, about 80% of patients with solid tumors and 40% of patients with malignant lymphomas, developed an adequate immune response to protect against influenza and pneumococcal disease (91). Immune responses in patients with solid tumors undergoing chemotherapy are higher than in hematologic malignancies. Adequate immune response in patients with lung cancer and breast cancer undergoing mild to moderate cytotoxic chemotherapy is observed in about 80% of patients (91, 92). Higher doses or repeated doses can be used to increase immunization by vaccines. Assessing the immune responses developed to understand the need for revaccination can be helpful (93). The results of studies that examine the association between recent cytotoxic chemotherapy and its consequences in cancer patients with COVID-19 are conflicting. Some studies confirm a link between cytotoxic chemotherapy and the severity of COVID-19 (18, 31), while others report no association (24, 29). The results of the study by Funakoshi et al. Showed that the level of anti-S1 antibody in cancer patients treated with cytotoxic chemotherapy was lower than the healthy group after the second dose (94). In addition, the results of several studies have shown that the level of neutralizing antibodies in cancer patients receiving active systemic chemotherapy is significantly lower than in healthy individuals (95, 96). Cytotoxic chemotherapy may reduce the effectiveness of vaccines by suppressing the immune system. However, further studies are needed to clarify the matter.

### Immune checkpoint inhibitors

Treatment with Immune checkpoint inhibitors increases the number of active CD8+ T cells that have the antiviral ability (97). Activated CD8 + T cells increase the levels of cytokines such as IL-6 and IFN-γ, which kill cancer cells (98, 99). Immune interstitial lung disease is a common and fatal side effect associated with treatment with Immune checkpoint inhibitors. The incidence of Immune interstitial lung disease varies from 2.5 to 10% depending on monotherapy or combination therapy with Immune checkpoint inhibitors (100). In patients with COVID-19, the most common cause of death is acute respiratory distress syndrome, which is seen by high levels of interleukin, interferon, and other cytokines (101). Cancer patients suffer from Immune interstitial lung disease due to the use of Immune checkpoint inhibitors. It is debatable whether COVID-19 causes serious consequences in these patients.

Checkpoint inhibitors pose a risk of safety-related side effects (IRAEs) depending on the type of treatment (102). Therefore, this type of cancer treatment raises concerns about vaccination. Importantly, vaccination can over-stimulate the immune response and increase IRAEs in cancer patients receiving this type of treatment. One study found that patients who used immunosuppressive inhibitors for treatment if they received the flu vaccine had an IRAE above 50% (103). However, the results of a recent study conducted by a group in Israel did not show an increased IRAEs in cancer patients treated with immunotherapy after receiving two doses of Pfizer/BioNTech vaccine compared with healthy individuals (104).

A 52-year-old woman was receiving immunotherapy 10 days after receiving the first dose of the Pfizer-BioNTech COVID-19 vaccine and was HBV positive. Because both immune checkpoint inhibitors and Covid-19 vaccines stimulate an immune response, an increased incidence of a wide range of complications can occur (105). Cytokine release syndrome (CRS) was observed in a cancer patient after vaccination. Although CRS symptoms are not observed after vaccination, CRS-related cytokines increase about 1.5 times. Therefore, an increase in cytokine levels is not enough to make a diagnosis of CRS and needs to be studied (106). Interestingly, a study found that BBIBP-CorV vaccine does not reduce the clinical efficacy of camrelizumab in cancer patients and does not increase the severe side effects associated with PD-1. Therefore, cancer patients can be vaccinated without discontinuing anti-PD-1 therapy (107).

So et al. Studies were performed on cancer patients undergoing anti-cancer treatment during vaccination. Of the total patients, 94.9% had solid malignancy and 88.5% were receiving anti-cancer treatment during vaccination. Three patients undergoing immunotherapy showed new side effects, including worsening of pre-existing grade 1 pruritus, grade 2 transaminitis, and grade 2 hypocortisolism. Patients receiving immunotherapy within 6 months of vaccination appear to be at a lower risk of developing any vaccine-related adverse events as well (OR 0.495 [95%CI 0.256–0.958]; p = 0.0037). Negative independent predictors of developing vaccine-related systemic adverse events include receiving chemotherapy within 28 days of vaccination (OR 0.373 [95%CI 0.221–0.629]; p < 0.001) (108). Interestingly, one study found that anti-S1 antibody levels were significantly lower in the Immune checkpoint inhibitors group. This was an unexpected result, as Immune checkpoint inhibitors is known to boost the immune system (94). It must be determined whether the suppression of antibody production is due to the effect of cytotoxic chemotherapy prior to Immune checkpoint inhibitors treatment or to something else.

### Surgery

During the COVID-19 pandemic, limitations, including hospital beds, delayed non-urgent (especially non-cancerous) surgeries. However, surgical strategies for cancer patients, including lung cancer, did not change. However, the challenge of assessing the risks and benefits of surgical delay based on the severity of the Covid-19 epidemic was evident. These interventions to determine the time of surgery after Quid-19 are associated with a mortality rate above 10% to 24% for all surgeries (109, 110). Increased mortality is seen in cancer patients who develop COVID-19 after surgery. This mortality rate after lung cancer surgery is between 40-50% (111).

## Limitation and challenges

COVID-19 vaccines have shown very promising results. BNT162b2 and mRNA-1273 mRNA vaccines are safe and very effective. However, delaying general vaccination and waiting for them to be scheduled for vaccination may reduce a person’s motivation to get vaccinated. One of the obstacles to a successful vaccination program is public reluctance to vaccinate due to misconceptions and various concerns about vaccine safety and efficacy. Vaccines intended for general vaccination must be able to induce an adequate response to infection even in immunocompromised individuals. A very important point to note is that once a drug has shown its appropriate effects in the general population, should also the safety and efficacy of the drug in susceptible populations be investigated. These vulnerable populations can include patients with chronic diseases or patients with weakened immune systems. Finite data have been obtained from trials on the safety and efficacy of existing vaccines for critically ill cancer patients undergoing treatment. Because cancer patients who were treated with immunosuppressive drugs during the previous 25 weeks were excluded from the study, and this study limitation has made many information unavailable.

Although the covid-19 vaccines cannot produce proper effects in patients with malignancies, the results are much more effective after receiving a booster dose. There are very few studies that determine the effectiveness and safety of existing vaccines in cancer patients. Additionally, there are no data in active cancer patients or patients receiving active treatment. Determining the time interval to receive the vaccine requires consideration of the type of treatment for cancer, the risk of progressive cancer, the risk of COVID-19, the state of the disease and co-morbidities (112–114). However, the importance of vaccination as a protective strategy for vulnerable people should be noted, although it seems necessary to examine other known measures as well.

## Conclusions

Extensive global efforts to produce Covid-19 vaccines have been able to reduce Covid-19 mortality. The protective nature of the available vaccines is the production of neutralizing antibodies that make the vaccinated person immune to the SARS-CoV-2 virus, however, the production of neutralizing antibodies in cancer patients is lower than in healthy individuals. It is gratifying that a high percentage of patients with solid tumors developed humoral and cellular immunity after vaccination. However, factors such as chemotherapy can suppress immune responses. But choosing the best possible time to vaccinate cancer patients who are undergoing treatment can reduce this limitation. Although Covid-19 vaccines are not significantly effective in patients with malignancies due to limited immune responses (the nature of the disease and the use of treatments that reduce B cells), beneficial results were looked in some of these patients after booster doses. Therefore extensive studies with a large statistical population in cancer patients, especially leukemia patients, can shed light on many unknowns.

## Author contributions

ATJ and ZHA contributed to conception and design of the study. AK and GAW organized the database. ZHA and HKHO wrote the first draft of the manuscript. ATJ, SAA, ASH, and TRA wrote sections of the manuscript. All authors contributed to manuscript revision, read, and approved the submitted version.

## Funding

This study was financially and morally supported by the Al-Mustaqbal University College, Babylon, Hilla, 51001, Iraq.

## Conflict of interest

The authors declare that the research was conducted in the absence of any commercial or financial relationships that could be construed as a potential conflict of interest.

## Publisher’s note

All claims expressed in this article are solely those of the authors and do not necessarily represent those of their affiliated organizations, or those of the publisher, the editors and the reviewers. Any product that may be evaluated in this article, or claim that may be made by its manufacturer, is not guaranteed or endorsed by the publisher.
